# Phenotypic and genotypic antimicrobial resistance correlation and plasmid characterization in *Salmonella* spp. isolates from Italy reveal high heterogeneity among serovars

**DOI:** 10.3389/fpubh.2023.1221351

**Published:** 2023-09-07

**Authors:** Sara Petrin, Massimiliano Orsini, Andrea Massaro, John E. Olsen, Lisa Barco, Carmen Losasso

**Affiliations:** ^1^Microbial Ecology and Microrganisms Genomics Laboratory, Istituto Zooprofilattico Sperimentale delle Venezie, Viale dell’Università, Legnaro, Italy; ^2^Department of Veterinary and Animal Sciences, Faculty of Health and Medical Sciences, University of Copenhagen, Frederiksberg, Denmark; ^3^Applied Chemistry Laboratory, Istituto Zooprofilattico Sperimentale delle Venezie, Vicenza, Italy; ^4^OIE and National Reference Laboratory for Salmonellosis, Istituto Zooprofilattico Sperimentale delle Venezie, Viale dell’Università, Legnaro, Italy

**Keywords:** *Salmonella*, whole genome sequencing, antimicrobial resistance, MIC, multidrug resistance, plasmids

## Abstract

**Introduction:**

The spread of antimicrobial resistance among zoonotic pathogens such as *Salmonella* is a serious health threat, and mobile genetic elements (MGEs) carrying antimicrobial resistance genes favor this phenomenon. In this work, phenotypic antimicrobial resistance to commonly used antimicrobials was studied, and the antimicrobial resistance genes (ARGs) and plasmid replicons associated with the resistances were determined.

**Methods:**

Eighty-eight Italian *Salmonella enterica* strains (*n* = 88), from human, animal and food sources, isolated between 2009 and 2019, were selected to represent serovars with different frequency of isolation in human cases of salmonellosis. The presence of plasmid replicons was also investigated.

**Results and discussion:**

Resistances to sulphonamides (23.9%), ciprofloxacin (27.3%), ampicillin (29.5%), and tetracycline (32.9%) were the most found phenotypes. ARGs identified in the genomes correlated with the phenotypical results, with *bla*_TEM-1B_, *sul*1, *sul*2, *tet*A and *tet*B genes being frequently identified. Point mutations in *gyr*A and *par*C genes were also detected, in addition to many different aminoglycoside-modifying genes, which, however, did not cause phenotypic resistance to aminoglycosides. Many genomes presented plasmid replicons, however, only a limited number of ARGs were predicted to be located on the contigs carrying these replicons. As an expectation of this, multiple ARGs were identified on contigs with IncQ1 plasmid replicon in strains belonging to the monophasic variant of *Salmonella* Typhimurium. In general, high variability in ARGs and plasmid replicons content was observed among isolates, highlighting a high level of heterogeneity in *Salmonella enterica*. Irrespective of the serovar., many of the ARGs, especially those associated with critically and highly important antimicrobials for human medicine were located together with plasmid replicons, thus favoring their successful dissemination.

## 1. Introduction

In Europe, salmonellosis is the second most common zoonosis, with 87,923 confirmed cases in 2019 (1). In spite of more than 2,600 identified *Salmonella* serovars (2), only three serovars, namely *S. enterica* serovar Enteritidis (*S. enteritidis*), *S*. Typhimurium and the monophasic variant of *S*. Typhimurium (MVST), accounted for more than 70% of the human cases (1).

Most of the human cases of salmonellosis result in self-limiting gastrointestinal diseases, which does not require treatment with antimicrobials. However, treatment is required, when systemic infections occur, and it is therefore a serious health problem, when strains of important zoonotic pathogens, such as *Salmonella*, become resistant to commonly used antimicrobials. Antimicrobial overuse and misuse in humans and animals for food production is the main cause for the increase in antimicrobial resistance (AMR) in *Salmonella* (3), while mobile genetic elements (MGEs) have played a major role in the rapid spread of resistance genes among *Salmonella* strains (4).

Control strategies against salmonellosis have been implemented in the European countries at primary production level, with the aim of reducing the incidence of target serovars, i.e., serovars which have been designated as ‘particularly relevant for public health’ (Commission Regulation (EU) No 2160/2003, 5). This strategy is however challenged by the fact that serovars that used to be less frequently isolated from human specimens are being detected with increasing frequency (5, 6), also in animal populations in which national control plans to control *Salmonella* prevalence had been implemented (7). Importantly, Annex III from the Commission Regulation (EU) No 2160/2003 underlined that resistance(s) to relevant therapies for human infections was an important criterion to define which serovars with public health significance should be considered targets for the reduction of *Salmonella* prevalence in breeders population (8).

While phenotypic susceptibility testing has informed on the current level of resistance in strains of *Salmonella*, it does not have the power to inform on the underlying mechanisms behind AMR in strains, nor the mechanisms by which AMR spreads in the population. Whole genome sequencing (WGS) has therefore become a valuable support to phenotypic susceptibility testing in surveillance of AMR, allowing detection of the major antimicrobial resistance genes (ARGs) circulating in zoonotic pathogens and the MGEs which contribute to spread of AMR. The current study investigated the correlation between phenotypic and genotypic resistance to antimicrobials in a selection of *Salmonella* strains, isolated from humans, animals and food in Italy and belonging to serovars associated with different frequency of isolation from human infections. The study aimed to analyze the presence of AMR genes and MGEs in strains with AMR, in order to determine the resistance genes and plasmids, which seemed to contribute to the spread of resistant isolates.

## 2. Materials and methods

### 2.1. Dataset description

Eighty-eight Italian *Salmonella enterica* isolates, belonging to 15 different serovars, were selected. The serovars were chosen to represent serovars which are frequently (F) and rarely (R) isolated from human infections in the European Union (EU) countries (1). The serovars which are frequently isolated from human infections were represented by *S. enteritidis*, *S*. Typhimurium and MVST, while rarely isolated serovars from human infections were represented by *S*. Derby, *S*. Dublin, *S*. Hadar, *S. infantis*, *S*. Kentucky, *S*. Livingstone, *S*. Mbandaka, *S*. Montevideo, *S*. Newport, *S*. Rissen, *S*. Senftenberg, and *S*. Thompson. The strains were collected spanning the years 2009–2019, from different Italian regions, and were isolated from animals, food and human sources. The selected strains are part of a broader collection maintained at the National Reference Laboratory for Salmonellosis at the Istituto Zooprofilattico Sperimentale delle Venezie (Legnaro, Italy) and Istituto Superiore di Sanità (Rome, Italy). Two strains were selected for each serovar for each source (human, animal, and food), with the exception of *S*. Dublin and *S*. Mbandaka, for which only one human isolate was available (Table 1). The strains were maintained at the Istituto Zooprofilattico Sperimentale delle Venezie (IZSVe), in cryobank tubes at −80°C, with preservative medium (Copan Diagnostics, CA, United States).

**Table 1:** Description of serovars, sources and number of isolates used in the study.

Serovar	Source	No. isolates
Derby	Animal	2
	Food	2
	Human	2
Dublin	Animal	2
	Food	2
	Human	1
Enteritidis	Animal	2
	Food	2
	Human	2
Hadar	Animal	2
	Food	2
	Human	2
Infantis	Animal	2
	Food	2
	Human	2
Kentucky	Animal	2
	Food	2
	Human	2
Livingstone	Animal	2
	Food	2
	Human	2
Mbandaka	Animal	2
	Food	2
	Human	1
Montevideo	Animal	2
	Food	2
	Human	2
MVST	Animal	2
	Food	2
	Human	2
Newport	Animal	2
	Food	2
	Human	2
Rissen	Animal	2
	Food	2
	Human	2
Senftenberg	Animal	2
	Food	2
	Human	2
Thompson	Animal	2
	Food	2
	Human	2
Typhimurium	Animal	2
	Food	2
	Human	2

### 2.2. Antimicrobial susceptibility testing

Antimicrobial susceptibility testing was performed as minimum inhibitory concentration (MIC) by broth microdilution method with Sensititre EUVSEC panel (TREK Diagnostics System). Results were interpreted according to European Committee on Antibiotic Susceptibility Testing (EUCAST) epidemiological cut-off values (ECOFFs; http://www.eucast.org). Multidrug-resistant (MDR) strains were defined as resistant to one drug in at least three different antimicrobial classes (9).

### 2.3. DNA extraction and WGS analysis

The *Salmonella* isolates were processed for DNA extraction and sequencing as already described in Petrin et al. (10). Briefly, after culturing, genomic DNA (gDNA) was extracted using a commercial column-based kit (QIAamp DNA Mini, QIAGEN, Valencia, CA), and purified gDNA was quantified with a Qubit 3.0 Fluorometer (Life Technologies, Waltham, MA). Libraries for WGS were prepared using the Nextera XT DNA sample preparation kit (Illumina, San Diego, CA) following the manufacturer’s instructions. High-throughput sequencing was performed with MiSeq Reagent kit v3, resulting in 251 bp long paired-end reads, or NextSeq High Output kit v2.5, resulting in 151 bp long paired end reads. Subsequent bioinformatics analyzes on raw reads were performed as previously described in Petrin et al. (11).

### 2.4. Genomic analyzes

To confirm the serovar., *in-silico* serotyping was performed using three different tools: MOST 1.0 (12) and SeqSero 1.0 (13) on raw data, and SISTR 1.0.2 (14) on assembled data.

Plasmid replicons were identified using blastn 2.7.1 (15) against PlasmidFinder 1.3 database [downloaded on 05/03/2018 (16)], while acquired antimicrobial resistance genes (ARGs) and chromosomal point mutations against ResFinder 3.0 and PointFinder databases, respectively [downloaded on 05/03/2018 (17)].

E-value thresholds were adjusted for each search depending on database size and were set as follows: 0.001 for plasmid replicons search, and 0.01 for ARGs search, respectively. All hits were required to have a 60% minimum coverage of the reference sequence found in the database, while the minimum required percentage of identity was 90% for plasmid replicons search, and 80% for ARGs search.

To further characterize plasmids that potentially contribute to the spread of antimicrobial resistance genes, only contigs longer that 200 bp were retained from the assemblies. Barrnap v0.9 (18) was used to mask ribosomal sequences on contigs. Contigs on which PlasmidFinder had identified a plasmid replicon were identified and collected to keep track of the incompatibility group(s) for each sample having putative plasmid(s).

A reference database containing plasmids from the taxa *Enterobacteriaceae* was built as follow:

1. Genebank (.gbk) format files for plasmids identified in taxa *Enterobacteriaceae* (Taxid 543) were downloaded from the NCBI nucleotide database;
2. only ‘complete sequence’ and ‘circular’ sequences were retained;
3. sequences were clustered using cd-hit v4.8.1 software (19) and setting 100% redundancy;
4. sequences were annotated with Plasmid Finder to search for the incompatibility group.

After building the reference database, blastN (15) was used to identify contigs that matched in the plasmid reference database with 90% identity and 90% coverage: if a contig matched with a plasmid in the reference database having an incompatibility group already identified by PlasmidFinder in that sample, the contig was retained and added to the contig identified by PlasmidFinder. All the contigs from one sample belonging to the same incompatibility group already identified by PlasmidFinder were concatenated by means of 150 bp Ns linkers. BlastN (15) was used to compare the resulting pseudomolecule for each incompatibility group with the plasmid reference database, in order to identify the best match (i.e., the match with the lowest e-value).

### 2.5. Conjugation assay, detection of plasmid replicon and antimicrobial resistance genes

In order to confirm the presence of ARGs on plasmids, for convenience reasons, two *Salmonella* isolates were chosen from those showing at least one ARG and a plasmid replicon on a putative plasmid from the *in silico* genomic analyzes. The transfer frequencies of *tet*A and *cat*A1 genes were investigated by conjugation experiments with nalidixic acid resistant *E. coli* 1816 as recipient strain. Donors and recipient strains were grown in Luria-Bertani (LB) broth for 24 h at 37°C. Then, a 1:50 dilution was prepared for each strain, and bacteria were grown at 37°C to a final OD_600_ 0.4. Five hundred μl of the donor strain was added to 4.5 mL of the recipient strain, and the bacterial suspensions were filtered using 0.22 μm filters (Merck Millipore) on MacConkey plates, pre-heated at 37°C for 1 h. After incubation for 18 h at 37°C, the filters were washed with 10 mL of physiological saline and vortexed to completely resuspend the cells. The cellular suspensions were centrifuged at 5000 rpm for 10 min, and the pellets resuspended in 1 mL of physiological saline after removing the supernatant. Serial dilutions were prepared, and 100 μL were plated on LB plates supplemented with nalidixic acid (50 mg/L) and chloramphenicol (50 mg/L) or nalidixic acid (50 mg/L) and tetracyclin (50 mg/L) to select for transconjugant colonies. The transfer frequencies were calculated as the number of transconjugants obtained per donor. Selected transconjugants colonies were transferred onto MacConkey agar plates to confirm they were *E. coli* colonies.

Identification of plasmid replicons from transconjugant colonies was performed by PCR-based replicon typing using the PBRT 2.0 kit (Diatheva, Fano, Italy), according to manufacturer’s instructions.

To screen for the presence of chloramphenicol resistance genes in transconjugant colonies, a multiplex PCR targeting *cat*A1, *cml*A1 and *flo*R genes was performed according to the protocol described in Guerra et al. (20) using *cat*A1, *cml*A1 and *flo*R forward and revers primers. PCR was performed in a final volume of 50 μL using 1X Buffer Taq Gold, 2 mM MgCl2, 400 μM dNTPs, 1 μM each primer, and 2,5 U Taq Gold (Life Technologies). Thermal cycling consisted of 95°C for 5 min, followed by 30 cycles (95°C for 30 s, 55°C for 30 s, 72°C for 40 s) and a final step at 72°C for 5 min. Finally, to screen for the presence of tetracycline resistance genes in transconjugant colonies, a multiplex PCR targeting *tet* genes was performed according to the protocol described by Ng et al. (21) using *tet*A, *tet*B and *tet*F forward and revers primers. PCR was performed in a final volume of 50 μL using 1X Buffer Taq Gold, 2 mM MgCl2, 200 μM dNTPs, 1 μM each primer, and 2,5 U Taq Gold (Life Technologies). Thermal cycling consisted of 95°C for 5 min, followed by 30 cycles (95°C for 30 s, 55°C for 30 s, 72°C for 30 s) and a final step at 72°C for 5 min. Amplicons were confirmed on a 2% agarose gel (Merck Life Science).

### 2.6. Statistical analysis

The data were statistically analyzed with RStudio (22) to generate plots, while graphical analysis was performed using the ggplot2 package (23). In order to evaluate the agreement between phenotypic and genotypic resistance, Cohen’s kappa statistics and value of p were calculated in RStudio using the vcd package (24). A kappa value between 0 and 1 is assigned and values ≤0 indicate no agreement; 0.01–0.20 none to slight agreement; 0.21–0.40 fair agreement; 0.41–0.60 moderate agreement; 0.61–0.80 substantial agreement; and values in the range of 0.81–1.00 indicate an almost perfect agreement (25).

## 3. Results

### 3.1. Phenotypic resistance to antimicrobials

Antimicrobial susceptibility test, performed with the EUVSEC panel, showed MIC values above the cut-off value to at least one antimicrobial molecule in 48 out of the 88 isolates. Results of MIC tests and definitions of susceptibility testing categories, according to epidemiological cut-off values, are reported in Supplementary Table S1.

Resistances to tetracycline, ampicillin, ciprofloxacin and sulfamethoxazole were common, with more than 20 resistant isolates each (Supplementary Table S1). None of the tested isolates showed resistance to ceftazidime, meropenem and tigecycline, while resistance to azithromycin was identified in one *S*. Rissen isolate, and resistance to cefotaxime was identified in one *S*. Derby and one *S. infantis* isolate.

**Figure 1:**
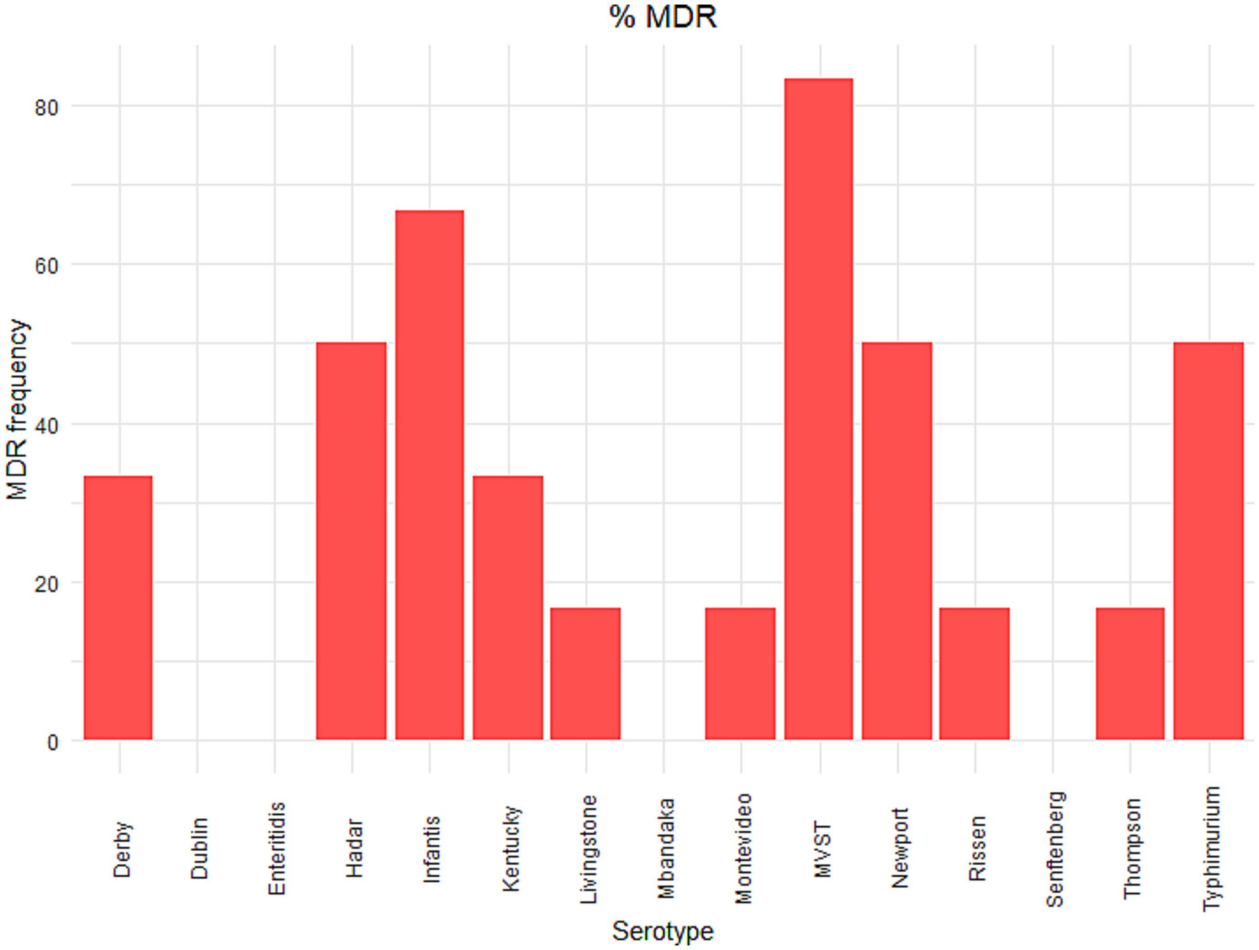
Phenotypic antimicrobial resistance in *Salmonella* serovars. The number of isolates resistant to antimicrobial molecules according to the European Committee on Antibiotic Susceptibility Testing (EUCAST) epidemiological cut-off values (ECOFFs; http://www.eucast.org). As described in Materials and Methods in the main text, two strains were selected for each serovar for each source (human, animal and food), with the exception of *S*. Dublin and *S*. Mbandaka, for which only one human isolate was available. Results of MIC tests and definitions of susceptibility testing categories, according to epidemiological cut-off values, are reported in Supplementary Table S1.

*S*. Senftenberg isolates did not show resistance to any tested drugs, while *S*. Dublin (3 out of 5 isolates) and *S. enteritidis* (3 out of 6 isolates) showed resistance to colistin only (Figure 1). Only one isolate of *S*. Mbandaka showed resistance to antimicrobials, and this isolate was resistant to ampicillin and ciprofloxacin (Figure 1). The other tested serovars showed resistance to different antimicrobial molecules (Figure 1). Five out of 6 isolates of *S*. Hadar showed phenotypic resistance to ciprofloxacin and tetracycline, and three of them to ampicillin and nalidixic acid. *S. infantis* showed resistance to ciprofloxacin, nalidixic acid and trimethoprim (4 out of 6 isolates), to sulfamethoxazole and tetracycline (3 out of 6 isolates), to ampicillin (2 out of 6 isolates) and one isolate showed resistance to cefotaxime.

Five out of 6 isolates of *S*. Kentucky showed phenotypic resistance to ciprofloxacin and nalidixic acid, and two of them, both isolated from human specimens, also resistance to ampicillin, gentamycin, sulfamethoxazole and tetracycline. Resistance to ampicillin and sulfamethoxazole was present in all the tested MVST isolates, and five out of six isolates showed phenotypic resistance also to tetracycline. Finally, four out of six *S*. Typhimurium isolates showed resistance to ampicillin, three isolates to sulfamethoxazole and tetracycline and two isolates also to chloramphenicol and ciprofloxacin.

**Figure 2:**
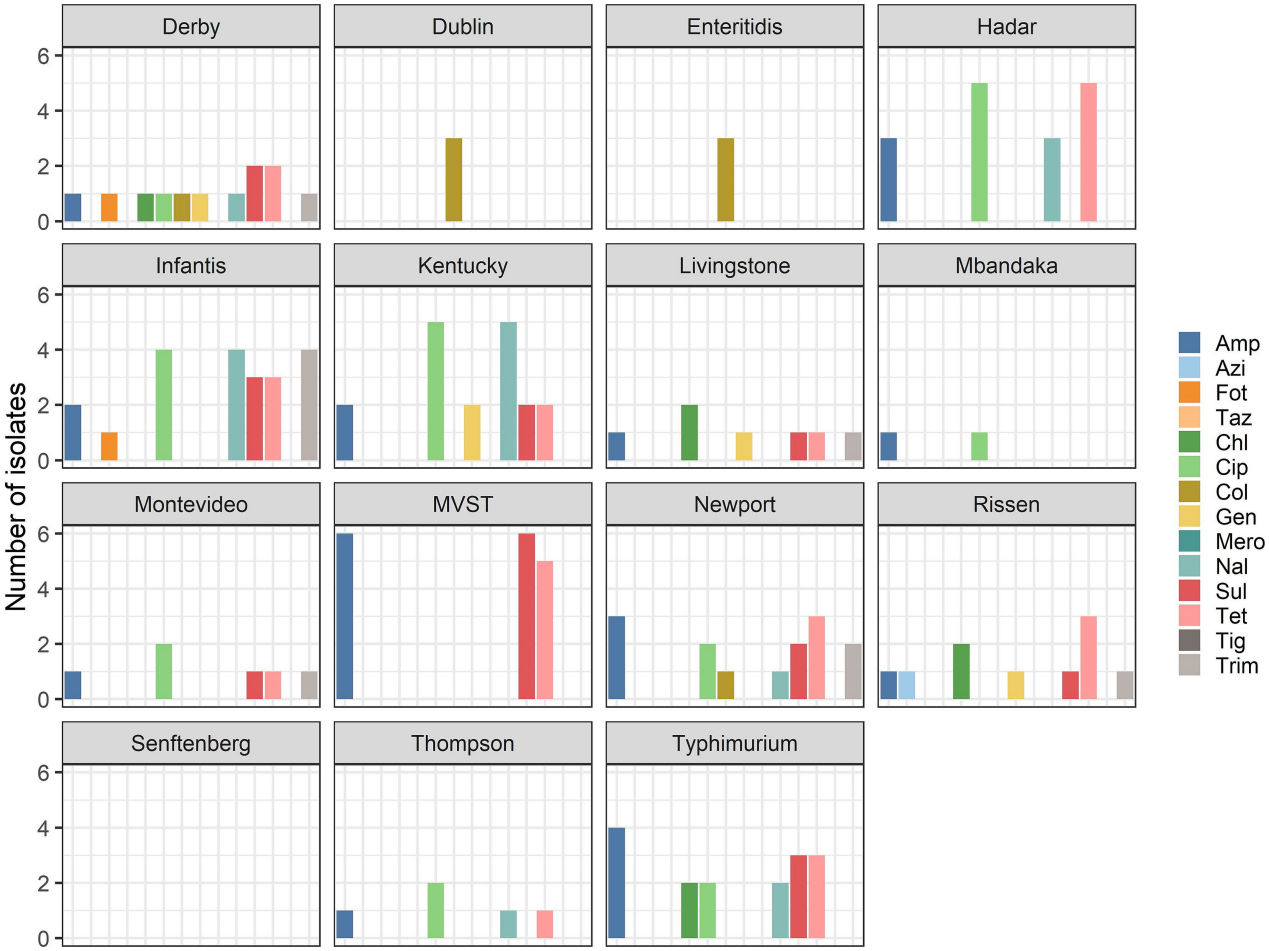
Percentage of multidrug-resistant isolates per serovar.

Of the isolates, which is a selection from a broader collection maintained at the National Reference Laboratory for Salmonellosis at the Istituto Zooprofilattico Sperimentale delle Venezie (Legnaro, Italy) and Istituto Superiore di Sanità (Rome, Italy), nine isolates from animals, nine isolates from food, and eight isolates from humans (25.9, 30.9, 33.3%, respectively) were multidrug-resistant. The percentage of strains within each serovar showing MDR are reported in Figure 2. The serovars with the highest number of MDR isolates were *S. infantis* and MVST.

### 3.2. Antimicrobial resistance genes

The dataset of genomic sequences was searched for the presence of known genes and chromosomal mutations conferring resistance to different classes of antimicrobials, and in total, 221 ARGs and AMR relevant point mutations were found (Table 1).

In details, 13 genes conferring resistance to aminoglycosides were identified and 6 genes conferring resistance to β-lactams. Among these, the most frequently identified ARGs to aminoglycosides were *aph(3″)-Ib* (*str*A) and *aph* (5)*-Id* (*str*B) identified in 19 genomic sequences, while the most common ARG to β-lactams was *bla*_TEM-1B_, found in 16 genomic sequences. The ARGs *sul*1 and *sul*2, conferring resistance to sulphonamides, were found in 11 and 13 genomic sequences, respectively. The ARGs *tet*A, *tet*B, *tet*G and *tet*D, conferring resistance to tetracyclines, were found in 18, 11, 2, and 2 genomic sequences, respectively. None of the selected samples presented *mcr* genes or mutation(s) in the chromosomal *pmr* genes, conferring resistance to colistin. When present, reduced susceptibility or resistance to (fluoro)quinolones was mainly caused by point mutations in *gyr*A (S83Y, D87N, D87Y, D87G, S83F) and *par*C (T57S) genes, while *qnr* genes were less commonly seen. Seven genomes were characterized by a point mutation in *gyr*A gene and a second mutation in *gyr*A, *gyr*B, or *par*C gene, while four genomes presented a point mutation in *gyr*A gene and *qnr* genes. This latter combination only confers reduced susceptibility to quinolones. Finally, genes conferring resistance to phenicol, trimethoprim, lincosamides and macrolides, and fosfomycin were present but in a limited number of genomic sequences (Table 2).

**Table 2:** Number of genomic sequences positive for ARGs and AMR relevant point mutations, divided by antimicrobial classes.

Antimicrobial class	ARG name^a^	Number of genomic sequences positive for the ARG^*^
Aminoglycosides	*aadA1*	1
	*aadA2*	5
	*aph(3″)-Ib (str*A*)*	19
	*aph(6)-Id (str*B*)*	19
	*aph(3′)-Ia*	5
	*aac(3)-IId*	2
	*aadA5*	1
	*aac(3)-IV*	1
	*aph(4)-Ia*	1
	*ant(3″)-Ia*	2
	*aac(3)-Id*	1
	*aadA7*	1
	*ant(2″)-Ia*	1
β-lactams	*bla*_TEM-1A_	1
	*bla*_TEM-1B_	16
	*bla*_CARB-2_	2
	*bla*_OXA-10_	1
	*bla*_CTX-M-1_	1
	*bla*_TEM-1D_	1
Phenicol	*cat*A1	3
	*flo*R	6
(Fluoro)quinolones	*gyr*A (S83Y)	3
	*par*C (T57S)	39
	*gyr*A (D87N)	3
	*gyr*A (D87Y)	1
	*gyr*A (S83F)	5
	*gyr*A (D87G)	4
	*qnr*B19	5
	*qnr*D1	2
	*qnr*S1	1
Sulphonamides	*sul*1	11
	*sul*2	13
Trimethoprim	*dfr*A1	1
	*dfr*A12	1
	*dfr*A17	1
	*dfr*A14	5
Lincosamides and macrolides	*lnu*G	1
	*mph*A	1
Tetracyclines	*tet*A	18
	*tet*B	11
	*tet*G	2
	*tet*D	2
Fosfomycin	*fos*A7	1

^a^Chromosomal point mutations conferring resistance to antimicrobials are reported in brackets. ^*^Some genomic sequences contained more than one ARG, conferring resistance to the same antimicrobial class.

A substantial agreement with statistical significance between genotypic predictions and phenotypic resistance to ampicillin (*k* = 0.79, *p*–value <0.01), azithromycin (*k* = 0.66, *p* = 0.03), chloramphenicol (*k* = 0.78, *p*–value <0.01), sulfamethoxazole (*k* = 0.78 *p*–value <0.01) and tetracycline (*k* = 0.64, *p*–value <0.01) was observed. Only fair agreement was found for ciprofloxacin (*k* = 0.35, *p*–value <0.01), nalidixic acid (*k* = 0.37, *p*–value <0.01) and trimethoprim (*k* = 0.40, *p*–value <0.01) resistance, while no to slight agreement was observed for cefotaxime (*k* = 0.04, *p*–value = 0.498) and gentamicin (*k* = 0.15, *p*–value = 0.06) resistance (Table 3).

**Table 3:** Comparison of phenotypic and genotypic characterization of 88 *Salmonella* isolates and results of the Cohen’s kappa statistics and value of p used to evaluate the agreement between phenotypic and genotypic resistance.

Antimicrobial class	No. of phenotypically resistant isolates	No. of genomes positive for at least a resistant gene/mutation^*^	Kappa	*p*-value	Antimicrobial class	No. of phenotypically resistant isolates	No. of genomes positive for at least a resistant gene/mutation^*^	Kappa	*P*-value
Aminoglycosides					Folate Pathway Inhibitors				
Gentamycin	5	29	0,15	0,06	Sulphonamides	21	22	0,78	<0,01
β-lactams					Trimethoprim	10	8	0,40	<0,01
Ampicillin	26	21	0,79	<0,01	Phenicols				
Cefotaxime	2	21	0,04	0,498	Chloramphenicol	7	8	0,78	<0,01
Fluoroquinolones					Tetracyclines				
Ciprofloxacin	24	53	0,35	<0,01	Tetracycline	29	31	0,64	<0,01
Nalidixic acid	17	53	0,37	<0,01	Macrolides and ketolides				
					Azithromycin	1	2	0,66	0,03

^*^For statistical purposes, the sum of ARGs conferring resistance to a specific antimicrobial compound was used to calculate the Kappa value. Ceftazidime, meropenem and tigecycline results are not reported since there were no phenotypically resistant isolates to these antimicrobials. Colistin was not reported since there were no antimicrobial resistance genes.

The distribution of ARGs per serovar showed that strains of *S*. Dublin and *S. enteritidis*, in agreement with their lack of phenotypic resistances which are not associated with point mutations, were without antimicrobial resistance genes (Supplementary Figures S1–S15). The distribution of ARGs per source revealed similar profiles in animal, food and human strains. The most frequently identified ARGs in the three sources were *sul1* and *sul2*, *tet*A and *tet*B, *aph(3″)-lb* (*str*A) and *aph* (5)*-Id* (*str*B), and the chromosomal point mutation in *par*C (T57S), potentially conferring reduced susceptibility to (fluoro)quinolons (Supplementary Figures S16–S18).

### 3.3. Plasmid replicons and co-location of plasmid replicons with ARGs

Among the 88 strains, 61 contained DNA sequences, which matched at least with one plasmid replicon (Supplementary Table S2). In total, 22 different plasmid replicons were detected, with Col(pHAD28), IncQ1 and IncFII(S) as the most frequently found (*n* = 20, *n* = 12 and *n* = 10 strains, respectively). In 20 sequences, at least two different plasmid replicons were detected and 5/20 contained IncX1 plasmid replicon. The frequency of detection of the plasmid replicon is reported in Table 4.

**Table 4:** Frequency of detection of plasmid replicons in strains of *Salmonella*, based on PlasmidFinder results.

Plasmid replicon name	Frequency of detection
Col(pHAD28)	20
Col156	3
Col3M	2
Col440I	6
ColE10	1
ColpVC	5
IncFIA(HI1)	1
IncFIB(pECLA)	2
IncFIB(pN55391)	3
IncFIB(S)	2
IncFII	1
IncFII(S)	10
IncHI1B(pNDM-CIT)	1
IncHI1B(R27)	1
IncHI2	0
IncHI2A	2
IncI1-I(Alpha)	3
IncN	4
IncQ1	12
IncX1	8
IncX4	1
IncY	1

Col(pHAD28) replicon plasmid was mainly found in *S*. Hadar (3/6) and *S*. Rissen (4/6) genomes, while IncFIB(pN55391) was only identified on *S. infantis* (3/6) genomes. IncX1 was predominantly found in *S*. Dublin (5/5) genomes, IncQ1 in MVST (6/6) genomes and IncFII(S) in *S*. Dublin (2/5), *S. enteritidis* (5/6) and *S*. Typhimurium (3/6) genomes (Supplementary Table S2).

In 14 genomic sequences, ARGs and at least one plasmid replicon were found to be located on the same contig, and only 3 strains carried plasmids with just one resistance gene [*tet*B (*n* = 1), *flo*R (*n* = 2)]. Detailed information about the co-occurrence of plasmid replicons and ARGs on contigs scored as plasmid contigs is reported in Supplementary Table S3.

### 3.4. Conjugation assay and confirmation of transconjugants determinants

Conjugation experiments using *E. coli* 1816 as recipient strain were successful and frequencies of conjugation were calculated as being 3.45 transconjugants per donor for a *S*. Newport strain resistant to tetracycline, and 1.48E-06 transconjugants per donor for a *S*. Livingstone strain resistant to chloramphenicol.

Twelve *E. coli* transconjugant colonies selected from LB plates supplemented with nalidixic acid and chloramphenicol showed the *cat*A1 gene amplicon and *tet*A gene was successfully amplified from sixteen *E. coli* transconjugant colonies selected from LB plates supplemented with nalidixic acid and tetracycline (Supplementary Figures S19, S20). The presence of IncN and IncHI2 plasmid replicons were confirmed in transconjugants from *S*. Newport and *S*. Livingstone strains, respectively.

## 4. Discussion

Antimicrobial resistance (AMR) occurs in microorganisms that become resistant to molecules intended to limit or prevent their growth, and it is considered a major threat to human and animal health (26, 27). In recent years, MDR has emerged as one of the most important threats to human health (28) and the spread of AMR is of particular concern in bacteria that represent common causes of infections in the human population, such as *Salmonella* spp. (29, 30).

Resistance levels in *Salmonella* vary by country, but on average 29,0%, 25,8% and 25,6% *Salmonella* isolates from human infections were reported to be resistant to sulphonamides, ampicillin and tetracyclines (30). The ability of *Salmonella* to acquire resistance genes from other bacteria is well described (4) and multidrug resistant (MDR) strains could cause infections that are more serious compared to those caused by pan-susceptible stains (28).

In this study, we characterized the phenotypic and genotypic antimicrobial resistance in a selection of Italian *Salmonella* isolates from human, food and animal sources. Moreover, WGS data were used to verify the co-occurrence of resistance gene and plasmids. Conjugation experiments confirming the plasmidic nature of ARGs were successfully carried out in two strains, where only one ARG and one replicon type were present, and transfer of resistance could firmly be linked to this MGE.

Only 48 strains among the 88 strains subjected to phenotypic testing were resistant to at least one antimicrobial, and among these isolates, 26 displayed MDR phenotype, with serovars *S. infantis* and MVST being the most MDR serovars. This level of MDR is similar to what has been reported at the EU level (1). In accordance with the reported data for the EU as a whole, MDR among Italian isolates was high among strains of MVST and *S. infantis*. Conversely, MDR is very high among isolates of *S*. Kentucky at the EU level [73.7% (31)], while the proportion was much lower in the studied samples, where only 33.3% of S. Kentucky isolates were MDR, probably reflecting the low number of strains analyzed in the current study.

Interestingly, the proportion of MDR isolates was found to be higher in isolates from food and human sources than from animals. This result opens to two possible scenarios: a) the ARGs stabilize in bacterial communities isolated from food handling environment, eventually reaching the final products. Indeed it is commonly recognized that meat from animals never treated with antibiotics could harbor antibiotic resistant bacteria, and b) other sources, for example, meat handlers or meat processing surfaces hosting resistant bacteria, could contribute to the ARGs stabilization in the bacterial communities in food processing environments (32). An alternative explanation could be the ban of antimicrobial as growth promoter in veterinary settings (33).

The proportion (33.3%), coherently with the Italian scenario, was slightly higher than what has been reported from the EU as a whole (31), and it was comparable to the proportion of MDR in food isolates (30.9%).

The degree of concordance observed between predictions of ARGs and resistance to a specific class of antimicrobial varied from no agreement, as in the case of colistin to substantial agreement, as in the case of ampicillin, chloramphenicol and sulfamethoxazole. Similar variability in agreement has been reported in other studies (34, 35). For those classes where agreement is high, surveillance for resistance by WGS of strains is a possibility, while care should be taken to base surveillance on this methodology for the classes with low agreement.

One possible explanation for low agreement between some resistance genes with the related phenotypes is a biological explanation. Indeed, ARGs could not be expressed due to the presence of weak or distant promoter or due to mutations in the promoter regions (36, 37). Alternatively, a technical explanation can be given: when the epidemiological cut-off values used to define whether an isolate is resistant or susceptible are higher than the resistance imparted by the resistance genes, isolates are classified as susceptible, as already described for *aad*A genes and streptomycin resistance (38).

Resistance to ampicillin was found in almost 30% of the isolates, and in 60% of these isolates, this was sustained by the presence of *bla*_TEM-1B_ gene. Most of the isolates bearing the *bla*_TEM-1B_ gene were of serovar MVST, *S*. Hadar, and *S*. Newport. Other *bla* genes were identified in genomes of isolates resistant to ampicillin. The TEM β-lactamase genes are usually carried by transposons (39) and found in plasmids (40), which increases the spread of this mechanism of resistance, posing a great concern for human health. Ampicillin is indeed classified as a critically important antimicrobial (CIA) by WHO (3), and the presence of *bla*_TEM_ gene characterized pandemic clones such as the multiresistant MVST circulating in the European countries since 2006 (41).

Only two strains showed phenotypical resistance to cefotaxime, a third-generation cephalosporin, classified as highest priority CIA, and interestingly both were of human origin. For just one of them, an *S. infantis* isolate, it was possible to identify a *bla*_CTX-M-1_ gene responsible for the resistance. Also in this case, it is interesting to report that clonal lineages of ESBL-producing MDR *S. infantis* emerged recently in Italy and other European countries, causing human infections (42–44). Other identified genes conferring resistance to β-lactam antimicrobials (*bla*_OXA_ and *bla*_CARB_) were only detected in few genomic sequences. Nonetheless, given they usually are carried by plasmids and other MGE (45), they could potentially be transferred to other enteric bacteria and limit the therapeutic treatments in case of severe human infections. On the genomic sequences from *S*. Derby, the second isolate phenotypically resistant to cefotaxime, we could not identify any resistance gene that confers resistance to cefotaxime (46). We could therefore hypothesize the expression of efflux pumps that contribute to resistance to cefotaxime in this case (47, 48).

Resistance to sulfamethoxazole was found in 24% of the tested isolates, most of which carried *sul*1 or *sul*2 resistance genes. These genes are indeed the most common in the analyzed genomic sequences, especially in serovars *S. infantis*, *S*. Typhimurium and its monophasic variant. It is interesting to note that nine strains showing *sul*2 gene located on plasmid contig were not phenotypically resistant to sulfamethoxazole. Further studies are needed to elucidate this finding.

Only five isolates (5.7%) were phenotypically resistant to gentamicin, however, ResFinder identified resistance genes [*aad*A2, *aad*A5, *aad*A7, *aac*(3)-Id, *aac*(3)-IId, *aph*(3′)-Ia, *aph*(3″)-Ib (*str*A), *aph*(6)-Id (*str*B), and *ant*(2″)-Ia] in only four of them. Multiple genes were found in the same isolate, as already reported by other studies (49, 50). For the isolate genomic sequence where we could not identify any resistance gene, the presence and expression of efflux pump, such as AcrD, can contribute to gentamicin resistance (51, 52). The agreement between genotypic and phenotypic resistance was indeed only slight. Moreover, there were different isolates in which resistance genes to aminoglycosides and streptomycin were identified (53), but which did not show phenotypic resistance to antimicrobials. It is possible that these isolates lack other components necessary to transfer an acetyl group that is required for the resistance mechanism of kanamycin, and further studies are needed to understand the lack of kanamycin resistance in these strains, as suggested by other authors (35). The most common detected resistance genes to aminoglycosides were *aph*(3″)-Ib (*str*A) and *aph*(6)-Id (*str*B) especially in genomes of *S*. Hadar and MVST serovars, while the resistance gene *aad*A2 was mainly identified in isolated from *S*. Derby and *S*. Typhimurium.

Aminoglycoside resistance genes are enlisted as current threats for human health, since they are commonly associated to ESKAPE pathogens (54). Finding these genes in *Salmonella* isolates highlights the need for an active surveillance of emerging resistances also in community associated bacteria.

Phenotypic resistance to ciprofloxacin and nalidixic acid was expressed by 27 and 19% of the isolates, respectively, while only a limited number of strains carried *qnr* resistance genes, a transferable resistance mechanisms responsible for reduced susceptibility to quinolones (55). Susceptibility to nalidixic acid co-occurring with resistance to ciprofloxacin was observed, corroborating the possible occurrence of plasmid mediated quinolone resistance mechanisms (30). One *S*. Mbandaka isolate carried a *qnr*S1 resistance gene, 2 *S*. Montevideo isolates carried a *qnr*D1 gene and five isolates a *qnr*B19 gene (*S*. Thompson (*N* = 1), *S*. Newport (*N* = 2) and *S*. Hadar (*N* = 2)). Interestingly, only one of the isolates carrying a *qnr* gene was of human origin and none of them belonged to *S. enteritidis*, which showed an increased proportion of resistant isolates in 2016 (56), nor to *S. infantis* or *S*. Kentucky serovars, in which resistance to (fluoro)quinolones is widespread (57). High level of ciprofloxacin resistance is observed in isolates with both *qnr* genes and chromosomal mutations, such as double substitutions in *gyr*A and a single substitution in *par*C genes, such in the case of the dominant clone of S. Kentucky ST198 in Europe (57, 58). Indeed, point mutations in DNA gyrase A (*gyr*A gene, position 83 or 87) and topoisomerase C (*par*C gene, at position 57) were present in different Italian isolates and also in the *Salmonella* genomes from different European countries, especially *S. infantis* and *S*. Kentucky sequences (58, 59). Of interest, a high number of genomes showed a point mutation at position 57 of *par*C gene (T57S, *n* = 39). Previous report showed that *par*C T57S is a spontaneous compensatory mutation, resulting in resistance to nalidixic acid but sensitivity to ciprofloxacin in *Salmonella* isolates (60, 61). However, accumulation of mutations in *gyr*A or *par*C genes, together with *par*C T57S, resulted in complete resistance to ciprofloxacin in different *Salmonella* serovars (62, 63). The resistance to quinolones has been widely reported in *Salmonella* serovars, especially in serovars frequently isolated from poultry sources such as *S. infantis* and *S*. Kentucky, probably due to the selective pressure exerted on the microbial communities of poultry farming where the use of quinolones as therapeutic are still present (64). As for aminoglycoside resistance genes, also the presence of mobile genes such as *qnr* (S and B) are ranked among the current threads having the potential to contribute to MDR in pathogens (54).

Resistance to trimethoprim, encoded by *dfr*A1, *dfrA*12, *dfr*A17, *dfr*A14 genes, was identified in 11,4% of the Italian isolates and this level of resistance has been confirmed at the European level, also in successful epidemic clones (65, 66). Interestingly, two isolates, which showed phenotypic resistance to trimethoprim, lacked resistance genes to the molecule. Further investigations are needed to explore the possibility of efflux pumps or other mechanisms that could explain this phenomenon. The resistance to trimethoprim was particularly common among isolates of *S. infantis*, as already demonstrated (66). This high level of strains carrying trimethoprim resistance genes is quite alarming as these genes are enlisted among the Rank I AMR genes contributing to MDR in human pathogens (54). This is of particular relevance in *Salmonella* as the serovar mainly displaying these genes is *S. infantis*, a serovar with high potential of causing severe infections in humans and well known to carry SGI and large plasmids harboring MRG cassettes (58, 67).

Resistance to chloramphenicol was sustained by *cat*A1 (*n* = 3) and *floR* (*n* = 6) genes in the Italian isolates, while at the European level also *cml*A1 gene was widespread. Chloramphenicol is not used for treatment of humans due to toxicity risks, however, this drug class is classified as highly important antimicrobial for human health (3). Epidemic clones of chloramphenicol-resistant *Salmonella*, such as *S*. Typhimurium ST313 in Africa (68), *S*. Typhimurium DT104 (69), and even *S. typhi*, have emerged and chloramphenicol resistance genes are often carried in plasmids, together with other genes conferring resistance to streptomycin, sulfonamides, and tetracyclines (70).

Tetracycline resistance was confirmed in 33% of the Italian isolates, where *tet*A and *tet*B genes were identified. While *tet*A was identified in different serovars, such as *S*. Hadar, *S. infantis*, *S*. Newport, *S*. Rissen, *tet*B was predominantly identified in *S*. Typhimurium and its monophasic variant. The reasons for this different occurrence are not known, however, multiple studies showed the presence of *tet*B in clinically relevant clone of *S*. Typhimurium and its monophasic variant (71–74). In the current study, *tetA* was shown to be present on a conjugative plasmid in a tetracycline resistant strain of *S*. Newport, and this plasmid transferred resistance with high frequency to a strain of *E. coli*, suggesting that such plasmid confer high ability of spread of resistance.

Interestingly, resistance to colistin was identified only in eight isolates, most of which were of serovars *S*. Dublin and *S. enteritidis*. None of the isolates showed relevant chromosomal mutations or acquired *mcr* genes. These serovars belong to group D *Salmonella*, which are characterized by a decreased susceptibility to colistin, due to the presence of abequose, the dideoxyhexose characterizing O-antigen epitope of this group (75, 76). Despite the increasing number of *Salmonella* isolates carrying *mcr* genes conferring resistance to colistin (77–81), and the diverse variants of *mcr* genes (82), we did not identify any *mcr* variant in the studied genomes.

Many of the resistance genes identified in the studied genomes are usually located on plasmids, that play a major role in evolution and horizontal gene transfer of bacterial antimicrobial resistance (83). Plasmid replicons were indeed detected in 69% of the genomic sequences and belonging to all the selected serovars. Of note, plasmid replicons (Inc groups) were in most of the cases associated with only one serovar., with the exception of IncFIB(S) and IncFII(S), that were identified in both *S. enteritidis* and *S*. Typhimurium and IncX1, that was identified in both *S*. Dublin and *S*. Kentucky. Previous reports demonstrated that certain serovars presented specific incompatible plasmids (83–86). We developed a workflow to map the plasmid replicons against known *Salmonella* plasmids, and all the identified replicon carrying contigs showed similarity to published plasmid sequences *Salmonella enterica* strains.

The resistance genes located on the same contig(s) as plasmid replicons were encoded on IncQ1 plasmid type, mainly harbored by genomes of the MVST. Interestingly, multiple resistance genes (*sul*2, *aph*(3″)-I and *aph* (6)-Ic) were identified on such IncQ1 contigs. IncQ1 plasmids are present in 4–12 copies/cell and have a size range from 8 to 14 kb (83) and were reported to carry *bla* genes (87) or the *sul*2-*str*A-*str*B cluster (88, 89). Of note, *bla* genes were not located on contigs carrying plasmid replicons, such as IncX plasmids, which usually carry resistance genes to β-lactams and quinolones (83). Similar IncX1 plasmids were already identified in *Salmonella* and *E. coli* strains. Interestingly, *tet* genes, usually found on plasmids, were not detected on IncQ1 plasmids. Recently, Oliva et al. (90) reported a novel IncQ1 plasmid carrying *tet* genes and postulated that recombination between a recipient IncQ1 plasmid and the *tet*R-*tet*A gene cluster had occurred. We did not search for recombination events nor genetic elements that could favor recombination, however it is worth noting that plasticity in bacteria genomes could likely mobilize such regions and contribute to the spread of plasmids with multiple resistance genes. IncQ1 contigs were found to match with plasmids already identified in *E. coli* and *K. pneumoniae* (91, 92).

Surprisingly, only a limited number of replicon containing contigs, with the exception of contigs found in MVST and *S*. Typhimurium genomes, were predicted to have ARGs. This however can be explained by the multiple mechanism by which antimicrobial resistance could arise in *Salmonella*. Besides horizontal transfer, also translocation from plasmids to chromosome has been described, creating clusters or antimicrobial resistance islands that are now regarded as an efficient means of resistance genes dissemination (67, 93, 94). Moreover, MGEs together with integrons, transposons and insertion sequences, favoring genetic recombination mechanisms, facilitate the accumulation on resistance islands (70, 95, 96). Another explanation could be that multiple resistance genes are carried on very large plasmids, as in the case of the pESI megaplasmid in *S. infantis* (42, 97). Such mega plasmids, with sizes ranging from 280 to 320 kb, unlikely would be completely assembled from short-read sequencing technology, such as used in the current study. It is therefore possible that plasmid replicon and antimicrobial resistance gene(s) would be identified in different contigs, hampering the association between plasmid and resistances (98).

The advent of WGS has enabled the prediction of AMR and antimicrobial resistance surveillance from genomic data alone (99), demonstrating high concordance between the presence of known ARGs or mutations and MIC of several antimicrobials (100). Despite the need to harmonize and standardize pipelines and databases, one of the most important advantage of WGS for AMR surveillance is the unprecedented level of detail in one assay, that made it possible also to define multidrug-resistance with great precision compared to phenotypic tests, allowed the description of current and emerging trends in AMR and allowed to trace specific allele profiles, rather than just phenotypic patterns by drug class (100).

## 5. Conclusion

*Salmonella enterica* represents an extremely heterogeneous species, and diseases caused by non-typhoidal *Salmonella* serovars vary considerably, with some serovars being significantly more prone to cause infections in humans. The reasons behind this are not completely understood, even if virulence mechanisms and genetic differences are believed to contribute to its success (101). In this paper, we described the variability in resistance genes and potential plasmids that characterize a set of Italian *Salmonella* isolates. Many of the identified genes, especially those that confer resistance to critically and highly important antimicrobials for human medicine were located together with plasmid replicons on contigs, which mapped to known plasmid sequences, and such plasmids can potentially favor in the spread and dissemination of ARGs. Indeed, genome plasticity, even more if associated to multidrug resistance, seems to be an important characteristic of successful *Salmonella* clones, regardless of the serovar.

## Data availability statement

The datasets presented in this study can be found in online repositories. The names of the repository/repositories and accession number(s) can be found at: [https://www.ncbi.nlm.nih.gov/bioproject/ and PRJNA817603].

## Author contributions

SP conceived the work, performed the experiments and wrote the manuscript. MO and AM performed the analyzes. JO, CL, and LB critically reviewed the manuscript. CL and LB concepted the work. All authors approved the final version of the manuscript.

## Funding

This research was supported by EU funding within the NextGenerationEU-MUR PNRR Extended Partnership initiative on Emerging Infectious Diseases (Project no. PE00000007, INF-ACT).

## Conflict of interest

The authors declare that the research was conducted in the absence of any commercial or financial relationships that could be construed as a potential conflict of interest.

## Publisher’s note

All claims expressed in this article are solely those of the authors and do not necessarily represent those of their affiliated organizations, or those of the publisher, the editors and the reviewers. Any product that may be evaluated in this article, or claim that may be made by its manufacturer, is not guaranteed or endorsed by the publisher.
