# Supplementary material for: Phenotypic and genotypic antimicrobial resistance correlation and plasmid characterization in Salmonella spp. isolates from Italy reveal high heterogeneity among serovars
Source: Front Public Health. 2023 Sep 7;11:1221351. doi: 10.3389/fpubh.2023.1221351 (PMC10513437; doi:10.3389/fpubh.2023.1221351)
Supplement: Supplementary file 1 [file Data_Sheet_1.docx]

**Supplementary Material**

**Supplementary Figures S1- S15.** Distribution of ARGs according to serotypes.


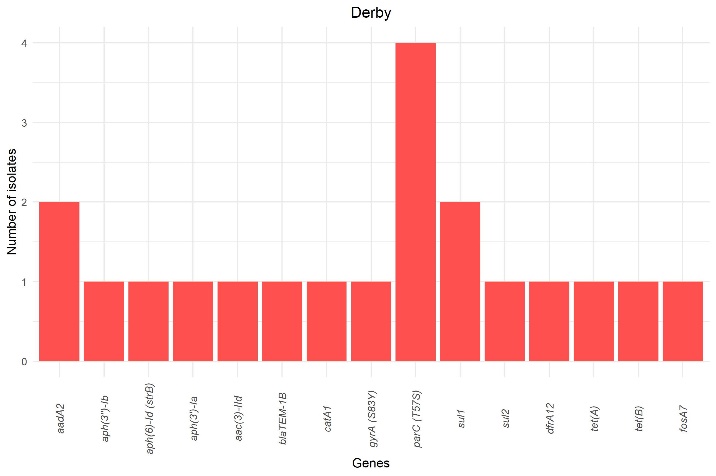

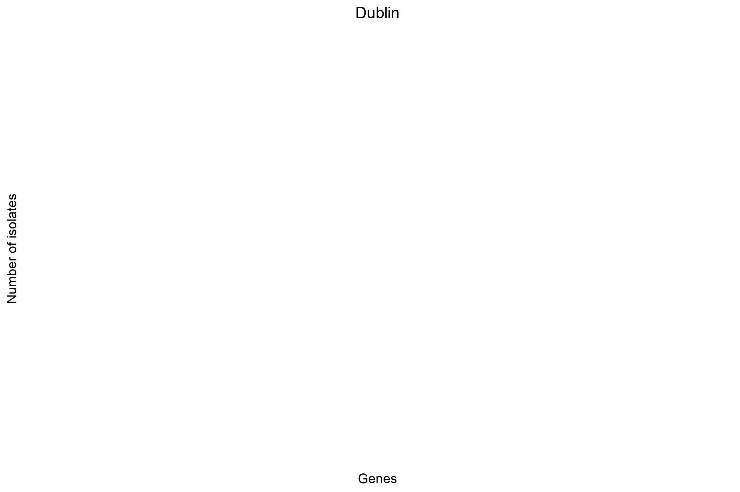

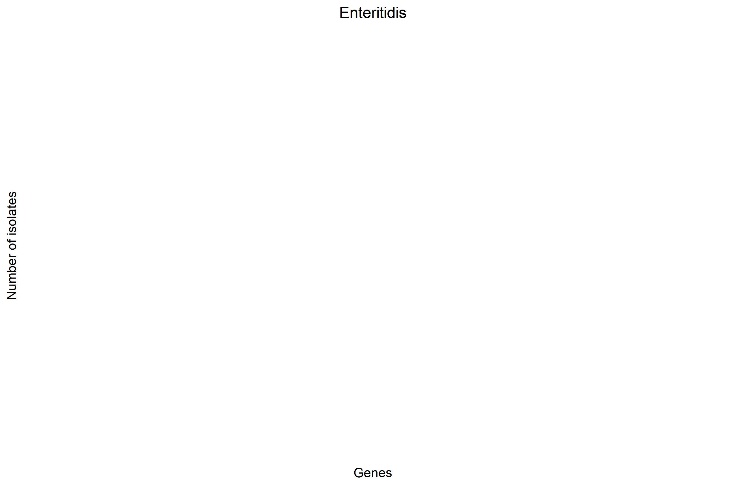

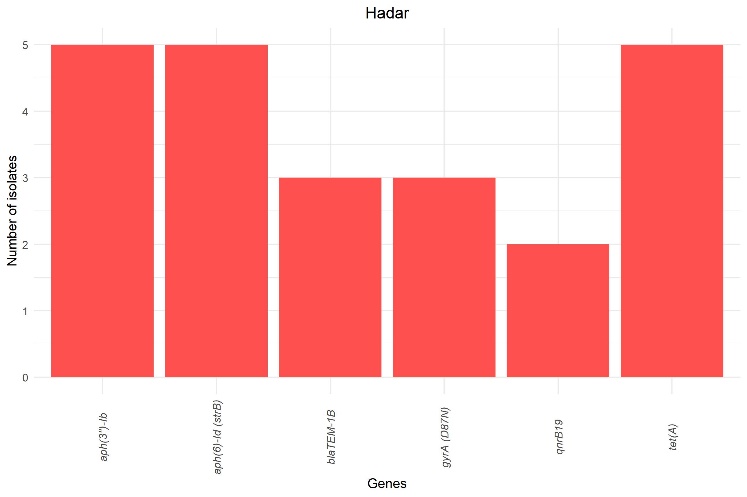

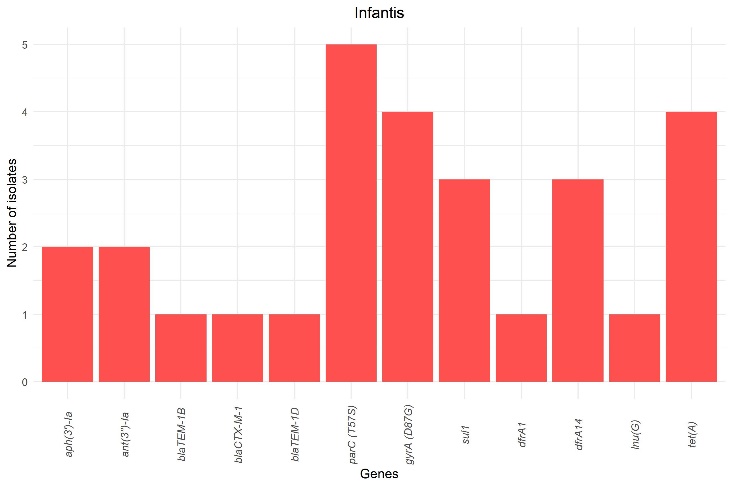

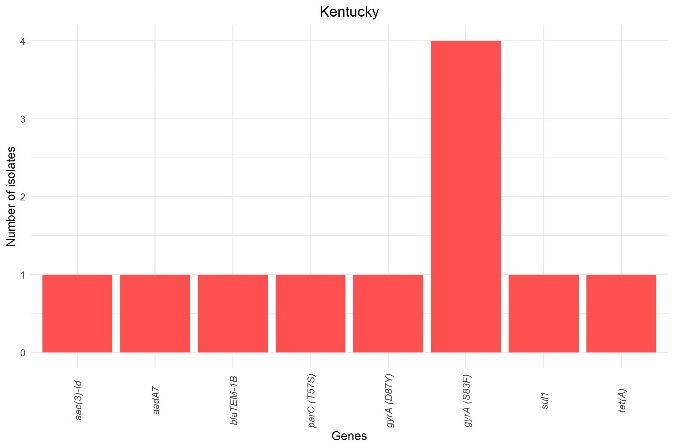

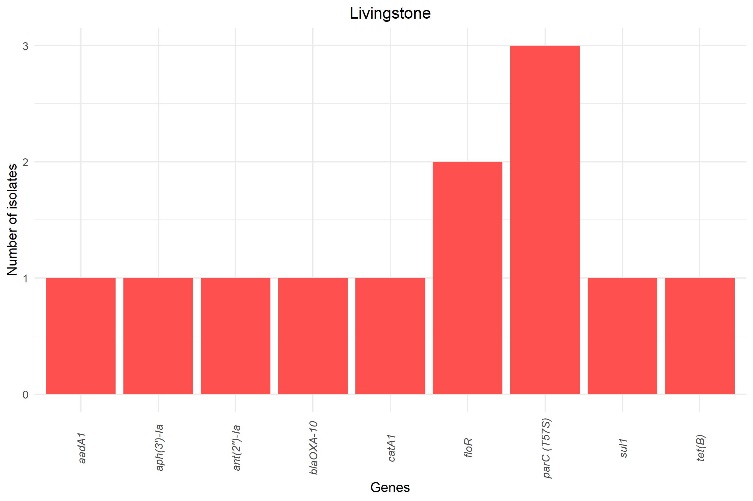

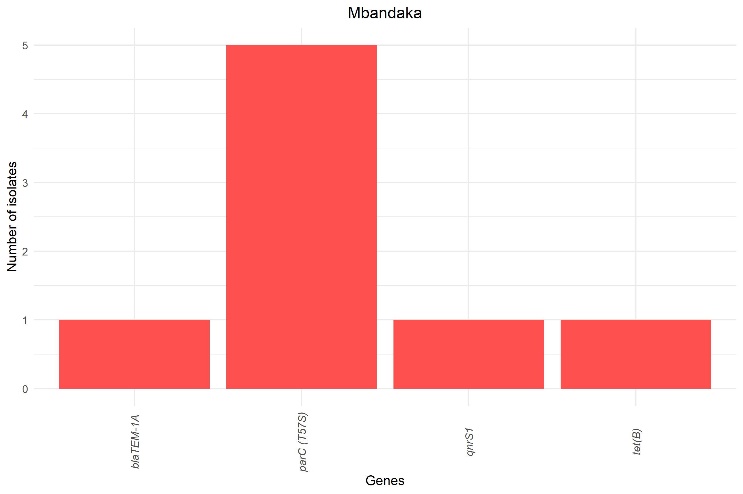

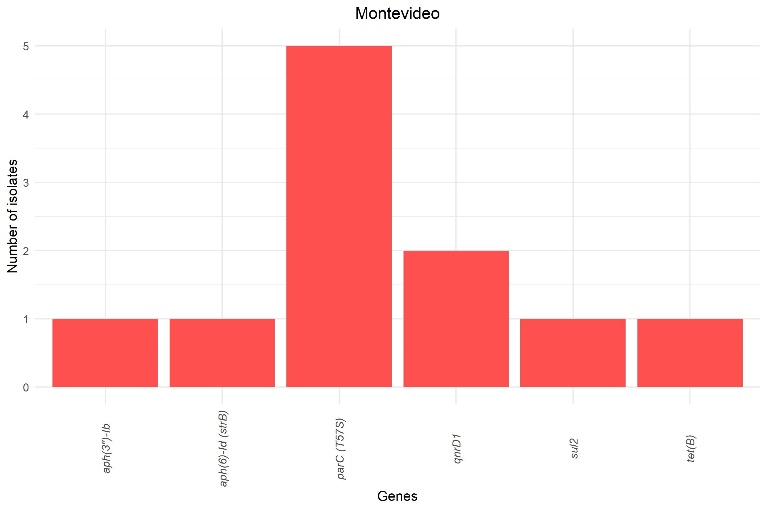

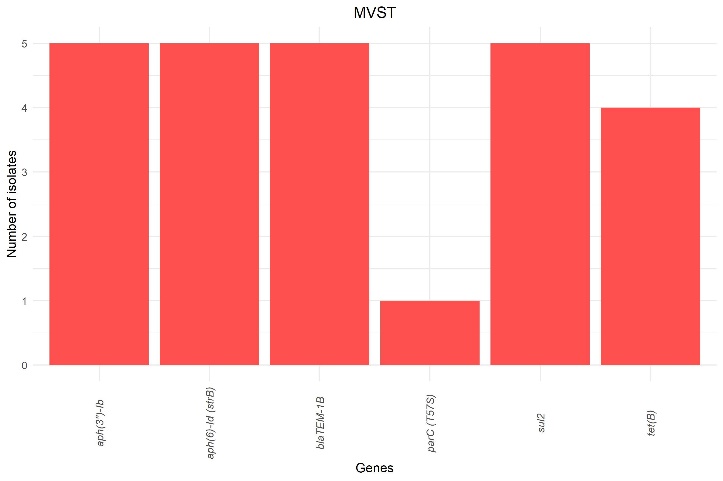

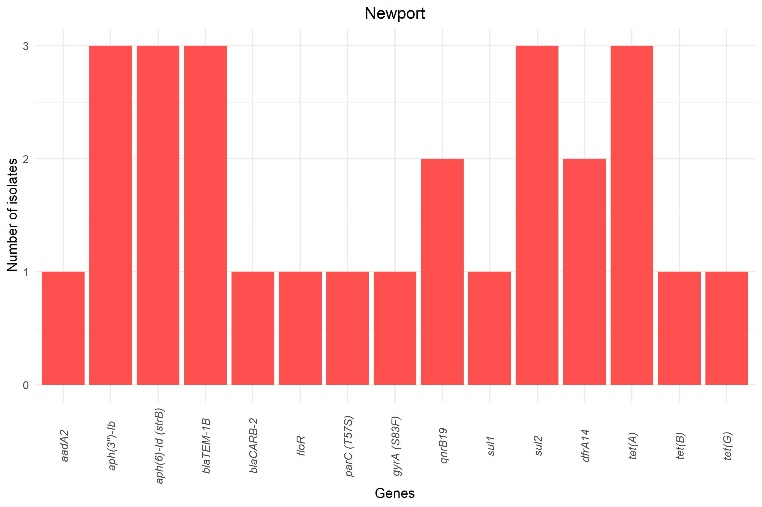

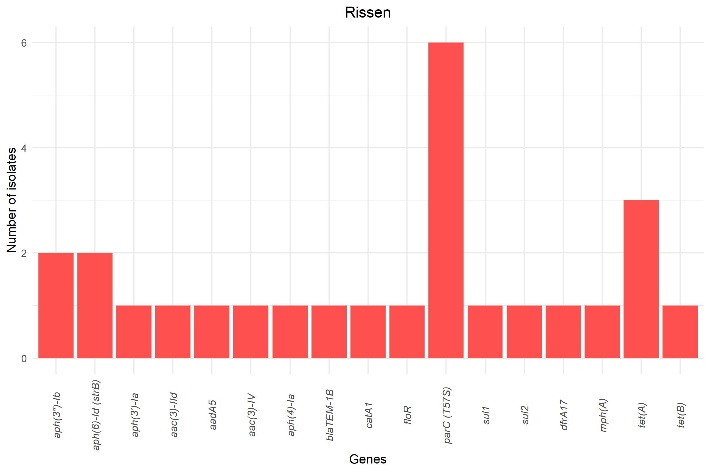

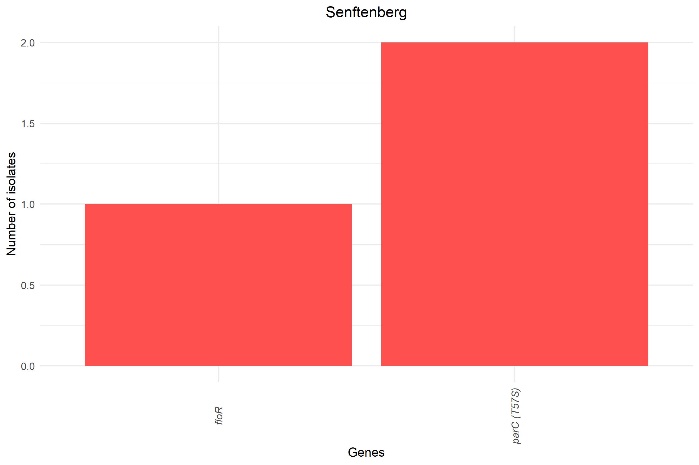

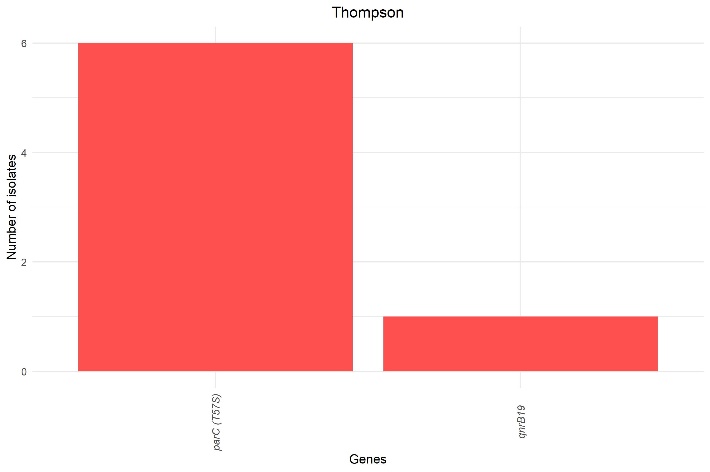

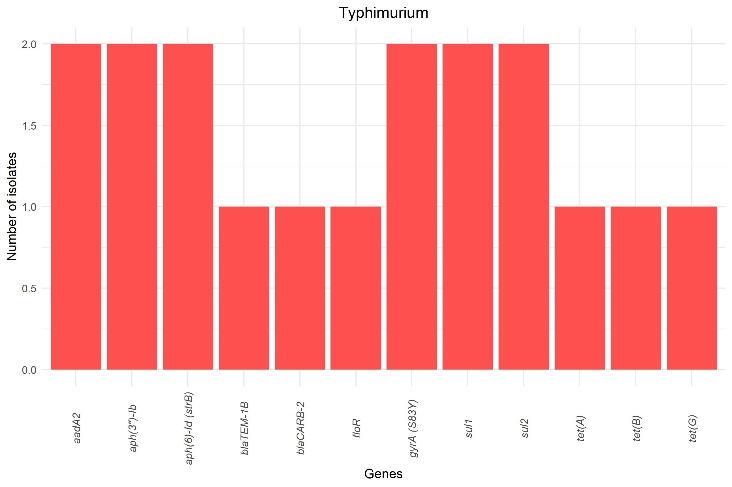


**Supplementary Figures S16- S18.** Distribution of ARGs according to source.


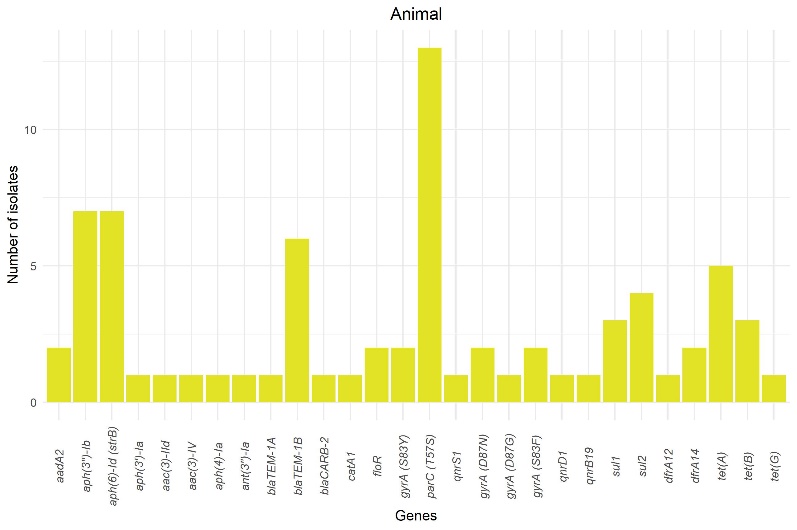

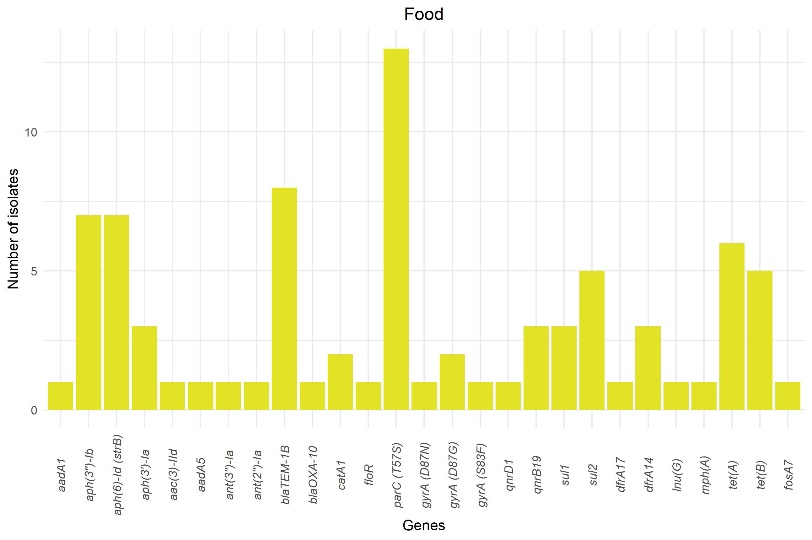

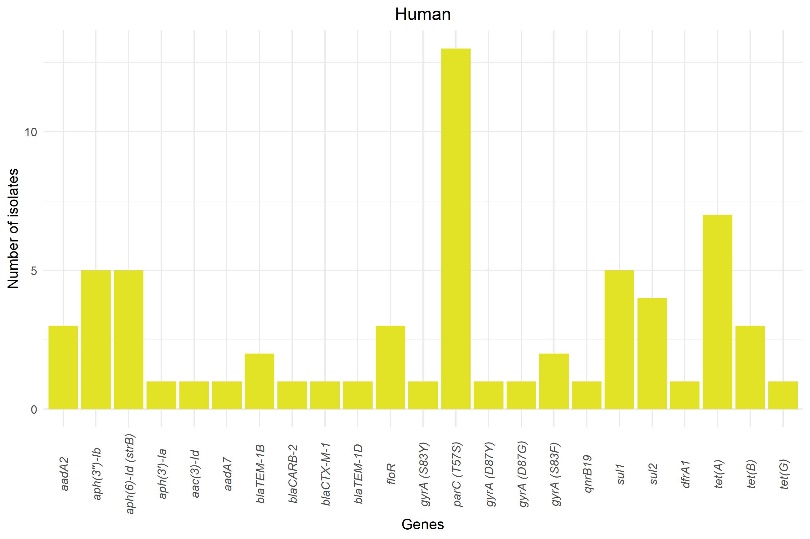


**Supplementary Figure S19**. Multiplex PCR for detection of *flo*R, *cat*A1 and *cml*A1 genes. Visualisation of the bands on an agarose (2%) gel for the twelve *E. coli* transconjugant colonies (lanes 1-12), donor strain (*S*. Livingstone 18SAL/239-9, lane 13), recipient strain (*E. coli* J53 R+, lane 13), positive controls and negative control. DirectLoad Wide Range DNA Marker (SIGMA) was used as molecular size marker. The size of each amplicon is indicated at the side.

**
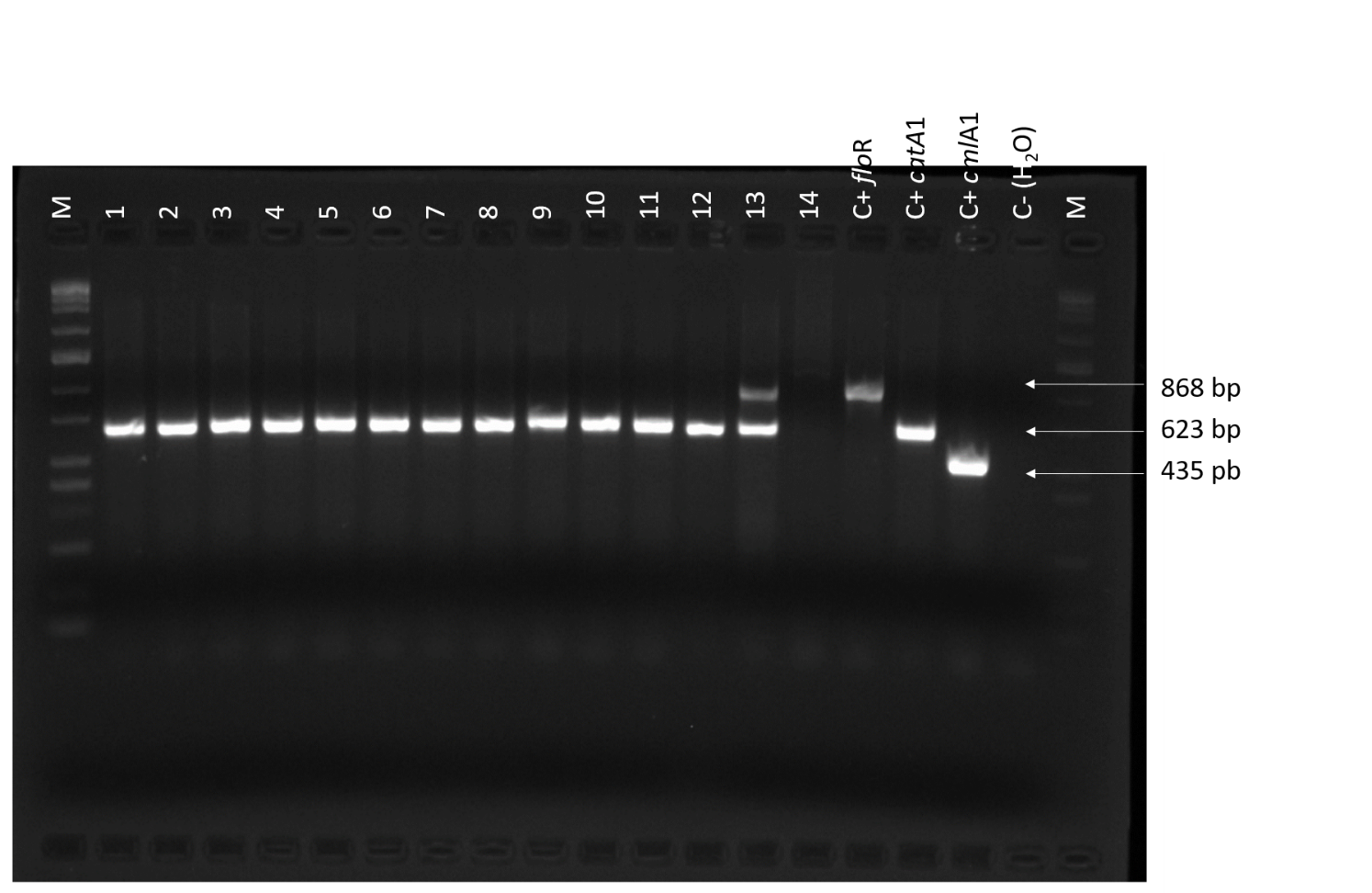
**

**Supplementary Figure S20**. Multiplex PCR for detection of *tet*A, *tet*B and *tet*G genes. Visualisation of the bands on an agarose (2%) gel for the sixteen *E. coli* transconjugant colonies (lanes 1-16), donor strain (*S*. Newport 17SAL/3172, lane 17), recipient strain (*E. coli* J53 R+, lane 18), positive controls and negative control. DirectLoad Wide Range DNA Marker (SIGMA) was used as molecular size marker. The size of each amplicon is indicated at the side.

**
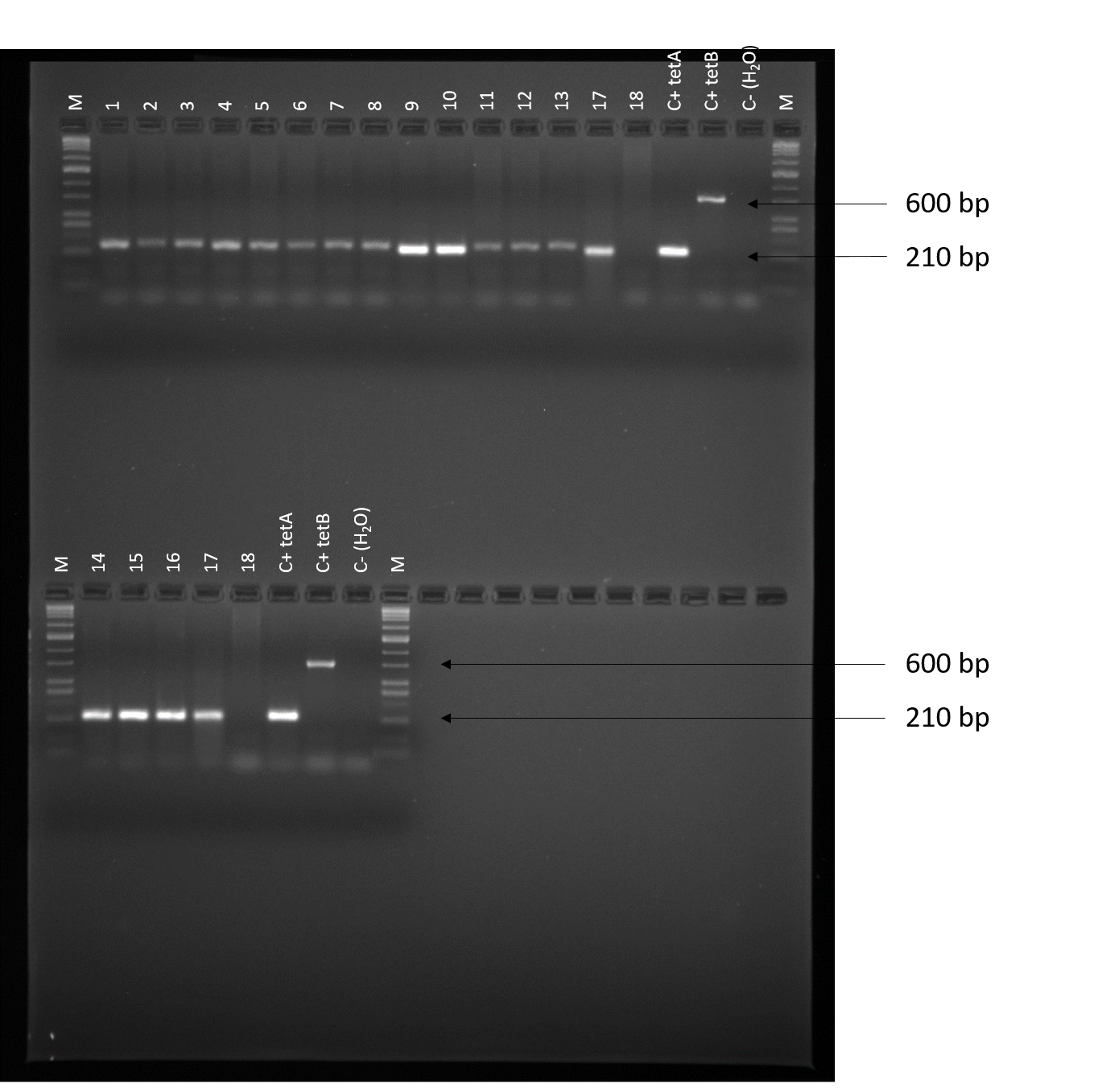
**
